# Is there reversible dimerization of albumin in blood plasma? And does it matter?

**DOI:** 10.1113/EP092012

**Published:** 2024-08-23

**Authors:** Gemma Harris, Michelle L. Bradshaw, David J. Halsall, David J. Scott, Robert J. Unwin, Anthony G. W. Norden

**Affiliations:** ^1^ Research Complex at Harwell, Rutherford Appleton Laboratory Didcot UK; ^2^ Department of Anaesthetics Bradford Royal Infirmary Bradford UK; ^3^ Department of Clinical Biochemistry Addenbrooke's Hospital Cambridge UK; ^4^ School of Biosciences University of Nottingham Sutton Bonington UK; ^5^ Department of Renal Medicine UCL Medical School London UK

**Keywords:** albumin, dimerization, fatty‐acid‐free albumin, plasma colloid osmotic pressure

## Abstract

Most albumin in blood plasma is thought to be monomeric with some 5% covalently dimerized. However, many reports in the recent biophysics literature find that albumin is reversibly dimerized or even oligomerized. We review data on this from X‐ray crystallography and diverse biophysical techniques. The number‐average molecular weight of albumin would be increased by dimerization, affecting size‐dependent filtration processes of albumin such as at the glycocalyx of the capillary endothelium and the podocyte slit‐diaphragm of the renal glomerulus. If correct, and depending on characteristics of the process, such as *K*_d_, reversible dimerization of albumin in plasma would have major implications for normal physiology and medicine. We present quantitative models of the impact of dimerization on albumin molecular forms, on the number‐average molecular weight of albumin, and estimate the effect on the colloid osmotic pressure of albumin. Dimerization reduces colloid osmotic pressure as total albumin concentration increases below that expected in the absence of dimerization. Current models of albumin filtration by the renal glomerulus would need revision to account for the dynamic size of albumin molecules filtered. More robust biophysical data are needed to give a definitive answer to the questions posed and we suggest possible approaches to this.

## Abstract (highlights)

- **What is the central question of this study?**
  Is there reversible dimerization of albumin in blood plasma? Albumin in blood plasma is generally considered monomeric apart from a small proportion of covalent dimer, but recent reports suggest it may actually be reversibly dimerized: is this physiologically significant?
- **What is the main finding and its importance?**
  There is some evidence that fatty acid‐free albumin dimerizes but less for native albumin. Dimerization, if proven, requires revision of the current molecular concepts underlying observed colloid osmotic pressures of plasma and renal ultrafiltration of albumin. Biophysical evidence for dimerization in plasma is currently weak.

## 1 INTRODUCTION

Most albumin in human blood plasma is thought to be monomeric with some 5% covalently dimerized (Al‐Harthi et al., 2019). However a recent review on protein oligomerization concludes as ‘fact’ that albumin undergoes concentration‐dependent reversible self‐oligomerization under physiological conditions (Kumari & Yadav, 2019). The data underlying this (Bhattacharya et al., 2014) are supported by many biophysical reports (Table 1), although reversibility has not always been explicitly established. The work discussed has used a variety of biophysical techniques studying human and bovine albumins as well as albumin which is ‘native’ and albumin‐made fatty‐acid‐free (FAF‐albumin). The results have been inconsistent quantitatively and we suggest possible reasons for this.

**TABLE 1:** Summary of biophysical studies of albumin dimerization.

Species	Albumin type	Solvent	°C	Albumin conc.	Method	$K_{d}$ dimer mol/L	Reversibility shown	Brief summary	Reference
Bovine	Fatty‐acid free (FAF)	PBS, pH 7.2	10	1–100 mg/mL	AUC: SE	4.5 × 10^−3^	Yes	Weak monomer/dimer (as stated) or no self‐association	Muramatsu and Minton (1989)
Human	‛Native’ and FAF	PB, pH 8.0	25	1–80 mg/mL	Osmometry, SAXS and DLS	—	No	FAF albumin, but not ‛native’ albumin demonstrated dimerization by all 3 techniques	Rescic et al. (2001)
Bovine	‛Native’ fluorescein‐, eosin‐labelled	MOPS, 10 mM, pH 5.8 (near pI)	25	Up to 3.3 mg/mL	Förster resonance energy transfer	10 ± 2 × 10^−6^	Yes	Energy transfer at steady‐state after 5 s indicating fast subunit exchange	Levi and González Flecha (2002)
Bovine	‛Native’	PB, pH 7.0	23	10–50 mg/mL	SAXS	—	No	Dimer observed at 50 mg/mL at pH 5.4 (near pI) but more than 85% monomer at other pH values	Barbosa et al. (2010)
Bovine	FAF	PB, pH 7.4	—	2–50 µmol/L	DLS	—	No	Self‐association increases from 50 to 150 µmol/L	Chatterjee and Mukherjee (2013)
Bovine and Human	FAF	PB, pH 7.4	—	2–150 µmol/L	CD and fluorescence spectroscopy	—	Yes	Intermolecular β‐sheet mechanism for self‐oligomerization	Bhattacharya et al. (2014)
Human	FAF	PBS, pH 7.4	—	2–150 µmol/L	Fluorescence and thioflavin‐T binding	—	No	Oligomer interfaces proposed to cause ‛intrinsic blue fluorescence’	Bhattacharya et al. (2017)
Bovine	‛Native’	Tris, pH 7.4 ionic strength 0.1 or 0.5	25–70	10–40 mg/mL	SAXS	Uncertain	No	Significant dimerization observed at >45°C	Molodenskiy et al. (2017)
Bovine	FAF	PBS pH 7.4	19.8	1.6–52 mg/mL	AUC: SV	No dimerization	N/A	See text	Chaturvedi et al. (2018)
Bovine	FAF^a^	50 mM Na‐phosphate pH 7.0		2.5–30 mg/mL	SAXS and neutron spin‐echo spectroscopy	See footnote^a^	No	Either interfacial contacts or covalent bond via Cys‐513 proposed dimerization mechanism	Ameseder et al. (2019)
Human	FAF	Deut. KPBS: Glycerol 1:1	<0	0.7−1.0 mM	Pulsed dipolar spectroscopy/EPR	0.1–1.0 × 10^−3^	Yes	Addition of myristate ‛decreased’ dimer conc.	Chubarov et al. (2020)

^a^ SAXS showed monomer:dimer‐ratio 1:2 over this conc. range, independent of concentration (pers. comun. Prof. A. Stadler, Jülich Centre for Neutron Science, Germany).

Abbreviations: AUC, analytical ultracentrifugation; CD, circular dichroism; DLS, dynamic light scattering; EPR, electron paramagnetic resonance; FAF, fatty acid free; PB, phosphate buffer; PBS, phosphate‐buffered saline; SAXS, small‐angle X‐ray scattering; SE, sedimentation equilibrium; SV, sedimentation velocity.

The distinction between studies of ‘native’ and FAF‐albumin is important because fatty‐acid loading causes a dramatic conformational change in albumin which may be relevant to dimerization (Curry et al., 1998).

If there is reversible concentration‐dependent dimerization or even oligomerization in plasma, implications for normal physiology have been suggested (Bhattacharya et al., 2014; Chubarov et al., 2020; Levi & González Flecha, 2002). Considering the movement of albumin from capillary vessels to interstitial fluid and then to lymph, each concentration change would be accompanied by a change in the monomer‐dimer equilibrium, Furthermore, dimerization would increase the number‐average molecular weight of albumin and change the reflection coefficient, σ, at the endothelial capillary. The reflection coefficient is the fraction of albumin molecules reflected by the membrane during a high rate of filtration. The molecular basis of the plasma colloid osmotic pressure (COP) and therefore Starling forces would be different from that solely due to monomeric albumin, and molecular theory underlying Starling forces would need revision. Protein filtration by the renal glomerulus would be affected in a similar way with a dynamic combination of monomeric and dimeric albumin being filtered. Current determinations of the glomerular sieving coefficient of albumin assume the protein is monomeric, so these would be in error. To simplify the presentation, unless stated, we only consider albumin self‐association to form dimers. The term ‘reversible’ is a convenient way of stating that albumin is weakly self‐associated ($K_{d}$ ≈ 0.01–1.0 mmol/L) and that equilibrium is established rapidly in relation to the kinetics of albumin concentration changes. To discuss the physiological implications of dimerization, we assume initially that the dimerization hypothesis is correct before further analysis. There are two main sources of underlying data on dimerization: X‐ray crystallography and biophysical studies in solution. However study of macromolecules such as albumin at the high physiological concentrations present in plasma ‘is a formidable experimental challenge’ (Chaturvedi et al., 2018).

## 2 DATA FROM X‐RAY CRYSTALLOGRAPHY

**FIGURE 1:**
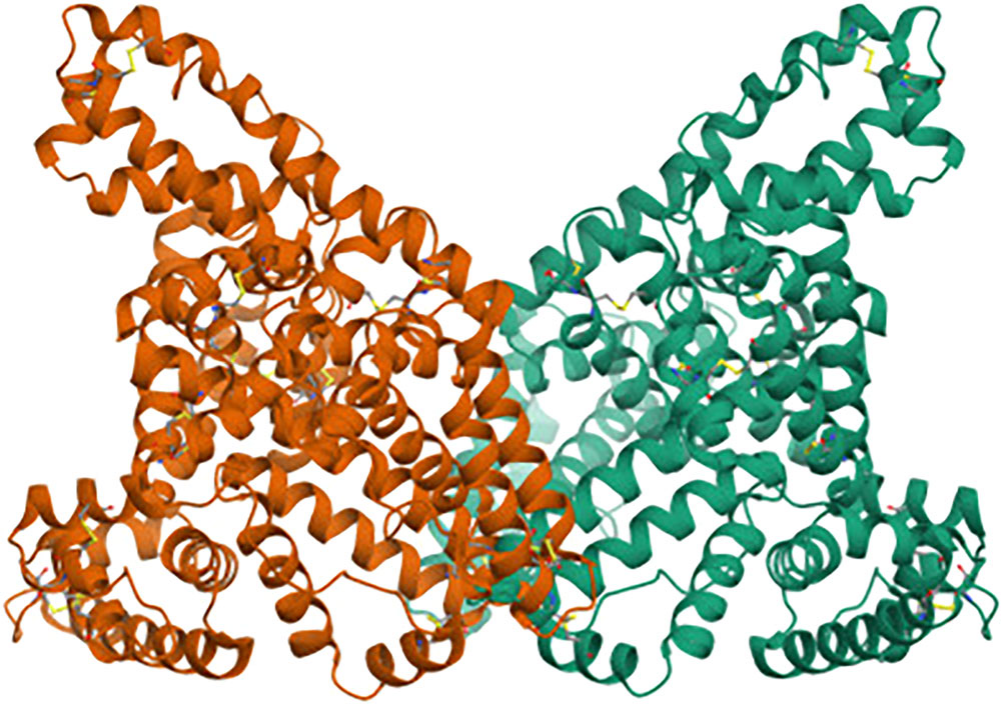
A crystal structure of fatty‐acid‐free (FAF) human albumin. Albumin from human plasma and recombinant albumin had virtually identical structures. The dimer is non‐covalent. https://www.ebi.ac.uk/pdbe/entry/pdb/1ao6/index. However, there is some evidence that this dimeric structure is an artefact of crystallization and not biologically relevant (see Appendix).

X‐ray crystallography demonstrates that crystals of both human (Figure 1) and bovine albumin may be dimeric (Bujacz, 2012; Sugio et al., 1999). Crystal structures show a dramatic conformational change between albumin that contains fatty acids and FAF‐albumin (Curry et al., 1998), which could be relevant both to interpretation of biophysical studies and to the physiological role of albumin. Caution is, of course, needed in extrapolating from crystal to solution structures (Mei et al., 2020) and at least one predictive structural tool suggests that the albumin dimerization cited may be an artefact of crystallization (see Appendix).

## 3 BIOPHYSICAL STUDIES OF DIMERIZATION IN SOLUTION

Table 1 summarizes, by publication date, studies most relevant to the question asked. We omit work using protein electrophoresis and size‐exclusion chromatography because over the time scales of these, reversible dimerization is expected to equilibrate dimers (Atmeh & Abuharfeil, 1993; Atmeh et al., 2007). The report by Lahiri et al. (2022) is excluded because it relies on the dimerization findings in two other reports included in Table 1 (Bhattacharya et al., 2014; Chubarov et al., 2020; Lahiri et al., 2022).

All studies reporting dimerization at pH values 7.0−7.4, and 37**°**C were obtained with fatty‐acid‐free albumin, but only four of the 12 studies cited examined ‘native’ albumins loaded with fatty acids. Sedimentation equilibrium studies were consistent with a $K_{d}$ value of approximately 4.7 mmol/L (Muramatsu & Minton, 1989). A more recent sedimentation velocity study found no evidence of self‐association but the novel approach used may nevertheless have difficulty identifying weak self‐association at high, non‐ideal, protein concentrations (Chaturvedi et al., 2018). None of the studies used physiological buffers and Ca^2+^ and Mg^2+^ were absent. No study except analytical ultracentrifugation explicitly accounted for sample viscosity as a possible confounder and no study provided kinetic information on dimerization. The on‐ and off‐rates for dimerization may be relevant to the kinetics of COP changes due to transients in fluid transport (Curry & Michel, 2021).

## 4 GENETIC STUDIES

If albumin dimerization is physiologically important, then one would expect mutations causing loss‐ or gain‐of‐function of dimerization to have a phenotype. This approach is difficult because the very rare complete loss of albumin in human, analbuminaemia, seems to have only a mild phenotype in adults with slight oedema, hypotension and fatigue (Minchiotti et al., 2019). Neonatal and childhood analbuminaemia is a risk factor for death. The likelihood of obtaining useful information relevant to dimerization is therefore small. In one family, connective tissue defects may be associated with albumin dimerization (Laurell & Niléhn, 1966; Weitkamp et al., 1973), but genetic information is generally uninformative regarding albumin dimerization.

## 5 IS FATTY‐ACID‐FREE ALBUMIN FOUND IN VIVO? AND HOW ‘NATIVE’ IS PURIFIED ALBUMIN?

How physiologically relevant are the large number of studies of fatty‐acid‐free albumin? And how relevant are native albumin studies given the extensive processing needed for purification?

Albumin is the major carrier of non‐esterified fatty acids (NEFA) for tissue energy production (Saifer & Goldman, 1961). Although NEFA levels vary widely with diet, weight and illness, typical mean fasting NEFA is 0.37 ± 0.16 mmol/L and the mean NEFA after a 75 g glucose load is 0.06 ± 0.05 mol/L (Oesterle et al., 2023). Since the great majority of NEFA are bound to albumin (Saifer & Goldman, 1961), this implies that a proportion of albumin molecules in plasma will be fatty acid free. Furthermore, albumin's role as a fatty acid transporter means that as NEFA are taken up into the interstitial spaces, it will become further depleted of fatty acids (van der Vusse, 2009). Complete loss of fatty acids may be unnecessary to induce the large conformational change between native and fatty‐acid‐free albumin (Curry et al., 1998). Partial depletion may be enough and in vivo dimerization of at least some albumin is then suggested by many of the studies in Table 1. Whether FAF‐albumin would form a hybrid‐dimer with fatty‐acid‐loaded albumin is an open question.

All studies have used commercial purified albumins and it is often difficult to obtain exact details of the processes used, but for human albumin pasteurization of serum at 60–65°C to inactivate potential viruses usually precedes purification. Fatty‐acid‐free albumin may be preferred for biophysical studies because it is less heterogeneous than native albumin but typically preparation requires acid treatment at pH 3 with charcoal (Chen, 1967). An ideal, as below, would be to detect dimerization in plasma itself, avoiding the uncertainties of purification and buffers.

## 6 DIMERIZATION

### 6.1 Molecular basis of COP and Starling forces

**FIGURE 2:**
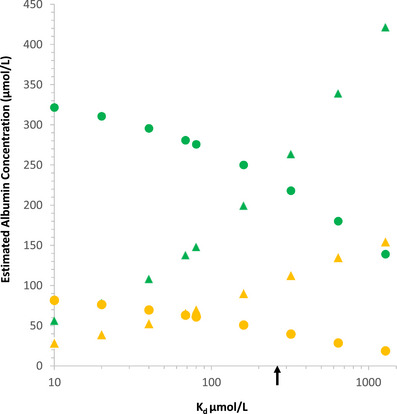
Estimated human albumin dimer (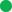) and monomer (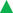) concentrations in plasma (45 g/L albumin) and dimer (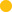) and monomer (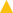) concentrations in the interstitial fluid of skeletal muscle (12.5 g/L albumin) with change of putative $K_{d}$ for dimerization in µmol/L. The scale for $K_{d}$ is logarithmic. Arrow for $K_{d}$ = 0.25 mmol/L corresponds to the $K_{d}$ values in Figures 3 and 4.

**FIGURE 3:**
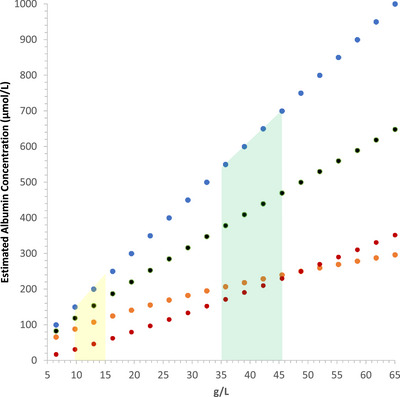
Human albumin dimer (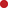), albumin monomer (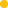) and monomer plus dimer (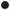) concentrations compared to the total albumin (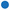) concentration over the normal and pathological range of plasma for dimer dissociation constant $K_{d}$ = 0.25 mmol/L. The green‐ and yellow‐shaded areas show normal ranges of the albumin concentration in plasma and interstitial fluid (skeletal muscle). See text for choice of $K_{d}$ = 0.25 mmol/L.

**FIGURE 4:**
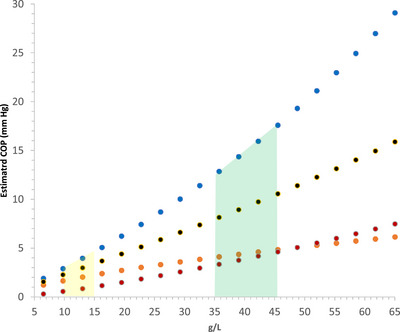
Estimated COPs due to human albumin dimer (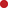), albumin monomer (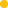) and monomer plus dimer (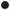) concentrations compared to measured total albumin COP (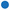) over the normal and pathological range of plasma for dimer dissociation constant $K_{d}$ = 0.25 mmol/L. The green‐ and yellow‐shaded areas show normal ranges of the albumin concentration in plasma and interstitial fluid of skeletal muscle (Ellmerer et al., 2000). Measured total albumin COP is from Landis and Pappenheimer (1963). See text for choice of $K_{d}$ = 0.25 mmol/L.

To understand the physiological consequences of dimerization we built a simple mathematical description of the molar concentrations of monomers and albumin dimers over the normal and pathological plasma range in humans (Appendix). Since biophysical data are currently inconsistent as to the $K_{d}$ for dimerization, we explore in Figure 2 the effects of varying this value over a range informed by the data in Table 1. This approach provides useful molar data for physiological and pathological plasma albumin mass concentrations. Figure 3 shows how the molar concentrations of albumin monomer and dimer vary over physiological concentrations for a $K_{d}$ of 0.25 mmol/L. This value for $K_{d}\;\mathrm{was}\;\mathrm{chosen}$ because it is fairly central within the very wide range of values in the literature (Table 1). At this value, monomer and dimer concentrations are approximately equal at an albumin concentration at the upper limit of the plasma reference range. This allows the predicted changes in concentration of both monomer and dimer to be clearly seen. Were the $K_{d}$ to be very much smaller, say 10 µmol/L, most albumin in plasma would be dimeric and conversely for a $K_{d}$ which is larger, more albumin would be monomeric. As well as molar concentrations, we are also interested in the effect on COPs. That is more difficult since using data from albumin solutions at physiological concentrations begs the question of the effect of dimerization. To bridge this gap and account partially for non‐ideality, we use the data for covalently dimerized albumin (Komatsu et al., 2004), an approximation since the putative reversible dimer may not have the same non‐ideality effects, including Gibbs–Donnan effects, as this covalent dimer. We further assume that pure albumin monomer will have double the colloid osmotic pressure of the dimer. In fact, the data reported for albumin solutions are about one‐half that of the dimer so we use that relationship (Komatsu et al., 2004; Landis & Pappenheimer, 1963). This allows an estimate of the total colloid osmotic pressure from monomer/dimer mixtures as outlined in the Appendix and presented graphically in Figure 4. For the reasons given, we have again used a hypothetical $K_{d}$ of 0.25 mmol/L. The results are at variance with the known, non‐ideal, change of colloid osmotic pressure of albumin solutions with concentration, also shown in Figure 4. A reason for this is apparent; as albumin concentration increases, a proportion of the additional molecules will associate as dimers so decreasing the proportionate increase in effective molecules and therefore colloid osmotic pressure. Experimentally the pressure actually increases more not less (Figure 4). This figure, of course, isolates the contribution of albumin and its possible dimerization to plasma COP and does not include that due to globulins (Landis & Pappenheimer, 1963) but the argument should be unaffected.

### 6.2 Filtration coefficients

**FIGURE 5:**
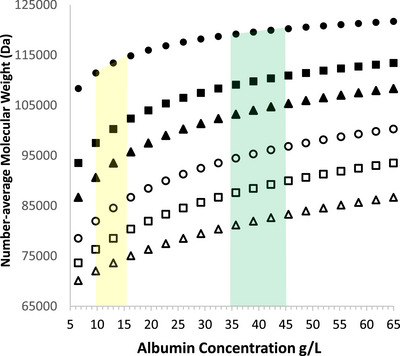
Number‐average molecular weights of albumin, calculated as in the Appendix, for dimerization $K_{d}$ values from 0.01 to 1 mmol/L. 0.01 (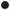), 0.05 (■), 0.1 (▲), 0.25 (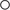), 0.5 (□) and 1.0 mmol/L (△). The green‐ and yellow‐shaded areas show normal ranges of the albumin concentration in plasma and interstitial fluid (skeletal muscle). △.

Dimerization decreases size‐dependent filtration coefficients and this can be assessed by the number‐average molecular weight, as outlined in the Appendix. Figure 5 shows this value, for several $K_{d}$ values derived from Table 1, plotted against the normal and pathological plasma albumin range in humans. The effect increases the number‐average molecular weight and decreases the filtration coefficient as concentration increases and this increases with smaller $K_{d}$ so that the apparent molecular wright may change from 70 to 120 kDa, considerably larger than the monomer, 65 kDa. Dimerization would decrease filtration expected at the capillary endothelial glycocalyx (Curry & Michel, 2021) and at the renal podocyte slit diaphragm (Ballermann et al., 2021) compared to lack of dimerization. In determining the glomerular sieving coefficient of albumin, a key parameter in renal physiology, albumin in plasma is taken to be monomeric (Ballermann et al., 2021). If there is, in fact, a dynamic mix of monomer and dimer (Figure 5) the estimate will be that for the unknown number‐average molecular weight and not the monomer, a criticism applying to our own work (Norden et al., 2001).

### 6.3 Translational physiology

Figure 4 shows that if albumin dimerizes in plasma, changes in albumin concentration may lead to smaller changes in plasma COP due to albumin than if dimerization is absent. COP would be ‘buffered’ as albumin concentration changes. However, the assumptions needed to derive albumin COP with dimerization and uncertainty over the $K_{d}$ for dimerization make these models imprecise and better biophysical data are needed. Buffering of albumin and plasma COP would be a novel physiological process but there are doubts that it actually occurs, as discussed above. Studies in the living dog do not support the concept of buffering (Prather et al., 1968). If it were correct, then there might be less reason for the use of albumin infusions in hypoalbuminaemic states because there would be partial compensation for the hypoalbuminaemia by dissociation of albumin dimers. Medical use of albumin has already been subjected to several critical appraisals (Featherstone & Ball, 2023)

Albumin's importance as a transporter of numerous drugs has suggested that dimerized albumin might offer better drug efficacy (Kinoshita et al., 2021; Komatsu et al., 2004). If a proportion of albumin is physiologically dimerized this would likely affect the pharmacokinetics of many drugs. Like renal glomerular transport of albumin, the molecular theory underlying the increased trans‐endothelial movement of albumin in acute inflammation would also need to be modified to understand the impact of the potential increase in apparent albumin molecular weight due to dimerization (Levick & Michel, 2010).

## 7 FUTURE APPROACHES

Solving the problems posed probably needs a combination of biophysical approaches. We suggest four: time‐dependent anisotropy decay (TDAD) studies either with or without a fluorescent label; further COP studies to compare FAF and native albumins and nuclear magnetic resonance (NMR). TDAD with a covalent label should provide quantitative information on albumin dimerization. The principle is that the rate of decay of fluorescence anisotropy depends on the hydrodynamic radius and therefore molecular weight of the protein studied (Lakowicz, 2006). Dimerized albumin will tumble more slowly in solution than the monomer, and signals from the two species can be measured and the proportion of dimer and monomer inferred. If a covalent fluorescent label is used, this approach requires that the label neither enhances nor inhibits dimerization and that labelling produces only a single labelled species of albumin. The fluorescent albumin monomer should equilibrate with dimer in the same way as the unlabelled monomer. Control experiments would provide reassurance that this condition is met. This approach would, in principle, allow studies in whole plasma as well as purified albumin since plasma could be ‘spiked’ with a fluorescent albumin which would equilibrate with endogenous albumin under relatively physiological conditions. This has the advantage over the studies in Table 1 that the bulk of the albumin is native, all globulins are present and endogenous plasma is used so avoiding the introduction of buffers. The lifetime of the fluorescent label should be comparable to the tumbling time of albumin to ensure that monomer and dimer albumin can be distinguished. Our own studies so far with long‐lifetime diazaoxatriangulenium dyes (Bora et al., 2016) have not met this criterion (unpublished observations) but longer‐lifetime ruthenium‐based adducts appear as attractive alternatives (Szmacinski et al., 1996). Such experiments need to control for the effect of viscosity on the tumbling times of proteins; high viscosity such as in plasma will increase tumbling time and this must not confound measurements of molecular weight.

Fortuitously human albumin has only a single tryptophan residue and its intrinsic fluorescence allows TDAD of purified albumin without using any label (Lakowicz & Gryczynski, 1992). This may be an attractive approach, at least for purified human albumins, and avoids the uncertainties of labelling artefacts.

If there is a difference between FAF‐ and native‐albumin in their tendency to dimerize, this should be apparent from COP measurements. Dimeric albumin will have a much lower COP per unit mass than the monomer and indeed a limited comparison of COP values between the two albumins over the albumin concentration range of 3–70 g/L supports this (Rescic et al., 2001). However the measurements of Rescic et al. (2001) were made in water at pH 5.8, a pH near albumin's isoelectric point, which favours dimerization since repulsive interaction due to charge is minimized. Performing a similar experiment using physiological buffers could provide direct evidence for or against dimerization

A direct method for investigating protein dimerization is NMR. Given the large size of albumin and its dimer, NMR is unfeasible at present. But rapid advances in this area should change this (Bax & Clore, 2019).

## 8 CONCLUSION

At present evidence for reversible dimerization of plasma albumin is weak. More robust biophysical data are needed to understand any physiological consequences. Until we have that data many of the ideas in the literature and presented here are speculative.

## AUTHOR CONTRIBUTIONS

Gemma Harris and David J. Halsall devised the TDAD experiments. David J. Scott critically reviewed analytical ultracentrifugation experiments. Gemma Harris undertook the ‘PISA’ analysis of albumin structures. Michelle L. Bradshaw suggested and reviewed translational implications of dimerization and Robert J. Unwin suggested several physiological implications of dimer formation. Anthony G. W. Norden wrote the initial MS. and all authors reviewed the MS. and made changes. All authors have read and approved the final version of this manuscript and agree to be accountable for all aspects of the work in ensuring that questions related to the accuracy or integrity of any part of the work are appropriately investigated and resolved. All persons designated as authors qualify for authorship, and all those who qualify for authorship are listed.

## CONFLICT OF INTEREST

None declared.

## FUNDING INFORMATION

None.

## Mathematical description of dimerization

Let $\mathrm{Al}b_{2}$ represent albumin dimer, Alb albumin monomer and [] be the molar concentration.

For the dimerization of albumin, $\mathrm{Al}b_{2}$ ⇌ 2 Alb the dissociation constant,

$$K_{d}={\left[\mathrm{Alb}\right]}^{2}/\left[\mathrm{Al}b_{2}\right]$$

(1)
$$\left[\mathrm{Al}b_{2}\right]={\left[\mathrm{Alb}\right]}^{2}/K_{d}$$

Let $\mathrm{Al}b_{\mathrm{tot}}$ be the total molar concentration of albumin molecules as monomer and dimer, so

(2)
$$\left[\mathrm{Al}b_{\mathrm{tot}}\right]=\left[\mathrm{Alb}]+2[\mathrm{Al}b_{2}\right]$$

[Alb] $=\;[\mathrm{Al}b_{\mathrm{tot}}]\;-$2[$\mathrm{Al}b_{2}$] $=\;[\mathrm{Al}b_{\mathrm{tot}}]\;-$2 ${\left[\mathrm{Alb}\right]}^{2}$/$K_{d}$, from (1)

2 ${\left[\mathrm{Alb}\right]}^{2}$ $+\;K_{d}$[Alb] $-\;K_{d}$ $[\mathrm{Al}b_{\mathrm{tot}}]\;=$ 0, in quadratic form and the real solution is

$$\left[\mathrm{Alb}\right]=(-K_{d}+\surd \left(K_{d}^{2}+8K_{d}\left[\mathrm{Al}b_{\mathrm{tot}}\right]\right))/4$$

This equation and Equations (1) and (2) were used in a Microsoft Excel spreadsheet to give Figures 2 and 3.

### Estimation of COP due to dimerization

Π_mono_ and Π_dim_ are defined as the COPs due to albumin monomer and dimer respectively.

An empirical equation for the variation of the COP of albumin is:

$$\Pi =2.8c+0.18c^{2}+0.012c^{3}\left(\mathrm{Landis}\;\&\;\mathrm{Pappenheimer},\;1963\right)$$

where *c* is the mass concentration of albumin in g/dL and converting to g/L.

(3)
$$\Pi =0.28c+1.8\times 10^{-3}c^{2}+1.2\times 10^{-5}c^{3}$$

where *c* is in g/L.

This is converted to µmol/L (to be consistent with Figure 3) by the relationship

$$\mu \mathrm{mol}/L=g/L\times 10^{-6}\times 65\times 10^{3}$$

where 65 × $10^{3}$ is the molecular weight of albumin monomer, so

µmol/L = 6.5 × $10^{-2}$ g/L. Expressing Equation (3) in µmol/L,

Π = 1.82 $\times \;10^{-2}$ *c* + 7.6 $\times \;10^{-6}$ *c* ^2^ + 3.3 $\times \;10^{-9}$ *c* ^3^ where *c* is in µmol/L

Following the argument in the section ‘Molecular basis of COP and Starling forces’, we take this COP as that due to the albumin monomer, so

$$\Pi_{\mathrm{mono}}=1.82\times 10^{-2}\;\left[\mathrm{Alb}\right]+7.6\times 10^{-6}{\left[\mathrm{Alb}\right]}^{2}+3.3\times 10^{-9}{\left[\mathrm{Alb}\right]}^{3}$$

where [Alb] is in µmol/L and the COP due to albumin dimer is estimated to be

$$\Pi_{\dim}=0.91\times 10^{-2}\left[\mathrm{Al}b_{2}\right]+3.8\times 10^{-6}{\left[\mathrm{Al}b_{2}\right]}^{2}+1.65\times 10^{-9}{\left[Alb_{2}\right]}^{3}$$

The data from Equations (1) and (2) give [Alb] and [$\mathrm{Al}b_{2}$] This gives Π_mono_, Π_dim_ and the total COP (Π_mono_ + Π_dim_) which were used in a Microsoft Excel spreadsheet to give Figure 4.

### Calculation of number‐average molecular weight due to dimerization

The number‐average molecular weight $M_{n}$ is defined as

$M_{n}\;=\;(\Sigma_{i}N_{i}M_{i}$)/$\Sigma_{i}N_{i}$ where $N_{i}$ is the concentration of the *i* th species in mol/L and $M_{i}$ is the molecular weight of that species (van Holde et al., 1998)

Applying this to a monomer/dimer mixture of albumin,

$$M_{\mathrm{mono}}=65,000\;\mathrm{and}\;M_{\dim}=130,000\;\mathrm{so}$$

$$M_{n}=\left(65,000\left[\mathrm{Alb}\right]+130,000\left[\mathrm{Al}b_{2}\right]\right)/\left(\left[\mathrm{Alb}\right]+\left[\mathrm{Al}b_{2}\right]\right)$$

Using the same approach as that to give Figure 2, a Microsoft Excel spreadsheet gives the values of $M_{n}$ in Figure 5 for $K_{d}$ values from 0.01 to 1.0 mmol/L.

### Review of literature of X‐ray crystal structures of albumin

To assess the biological significance of the dimeric albumin structures cited, we used the ‘Protein interfaces, surfaces and assemblies’ service (PISA) at the European Bioinformatics Institute (http://www.ebi.ac.uk/pdbe/prot_int/pistart.html), This ‘PISA’ analysis suggested that the dimeric structures cited may be an artefact of crystallization. We also searched for all structures in the RCSB Protein Database with ‛serum albumin’ given as the polymer entity description and performed a similar search in PDBePISA. This gave 185 structures in total. Of those, only two structures contain human albumin that is most probably dimeric (3JQZ and 6JE7) and two more where it is possible that the albumin is dimeric (one is human albumin 5IJF, one is ovine albumin 6HN0). These are all FAF‐albumin and 6JE7 is the only structure without a ligand bound. So, caution is needed as to whether the two dimeric X‐ray crystal structures cited correspond to the structures in solution, and the comprehensive search performed gives little support to the existence of non‐covalent albumin dimers in solution.
